# Exposure to Dengue Envelope Protein Domain III Induces Nlrp3 Inflammasome-Dependent Endothelial Dysfunction and Hemorrhage in Mice

**DOI:** 10.3389/fimmu.2021.617251

**Published:** 2021-02-25

**Authors:** Te-Sheng Lien, Der-Shan Sun, Cheng-Yeu Wu, Hsin-Hou Chang

**Affiliations:** ^1^ Department of Molecular Biology and Human Genetics, Tzu-Chi University, Hualien, Taiwan; ^2^ Center for Molecular and Clinical Immunology, Chang Gung University, Taoyuan, Taiwan

**Keywords:** dengue hemorrhagic fever, dengue envelope protein domain III, two-hit dengue mouse model, endothelial damage, Nlrp3 inflammasome, pyroptosis, necroptosis, apoptosis

## Abstract

Typically occurring during secondary dengue virus (DENV) infections, dengue hemorrhagic fever (DHF) causes abnormal immune responses, as well as endothelial vascular dysfunction, for which the responsible viral factor remains unclear. During peak viremia, the plasma levels of virion-associated envelope protein domain III (EIII) increases to a point at which cell death is sufficiently induced in megakaryocytes *in vitro*. Thus, EIII may constitute a virulence factor for endothelial damage. In this study, we examined endothelial cell death induced by treatment with DENV and EIII *in vitro*. Notably, pyroptosis, the major type of endothelial cell death observed, was attenuated through treatment with Nlrp3 inflammasome inhibitors. EIII injection effectively induced endothelial abnormalities, and sequential injection of EIII and DENV-NS1 autoantibodies induced further vascular damage, liver dysfunction, thrombocytopenia, and hemorrhage, which are typical manifestations in DHF. Under the same treatments, pathophysiological changes in the Nlrp3 inflammasome–deficient mice were notably reduced compared with those in the wild-type mice. These results suggest that the Nlrp3 inflammasome constitutes a potential therapeutic target for treating DENV-induced hemorrhage in DHF.

## Introduction

Dengue virus (DENV) infection is one of the most rapidly growing mosquito‐borne infections (1, 2). DENV infection is self-limiting. However, secondary DENV infections are epidemiologically associated with an increased risk of life-threatening severe dengue [classically known as dengue hemorrhagic fever (DHF)]. No specific antiviral treatments against DENV infection have been developed. Although overall mortality rates from infectious diseases decreased from 2005 to 2015, mortality attributable to DENV infection increased by 48.7% (3). Endothelial damage and vascular leakage are the hallmarks of severe dengue. Vascular leakage, which occurs during the acute phase of DHF, typically manifests 3–6 days after disease onset (4, 5). The fact that DHF acute phase follows the viremia-decreasing period and convalescence, suggests that it is DENV-induced endothelial injury, rather than infection of the endothelium, that contributes to such pathogenic responses (5). However, the viral factor responsible for vascular endothelial damage remains unclear.

The DENV envelope protein (E) domain III (EIII) is an Ig-like domain that is involved in host cell receptor binding for viral entry (6). However, cellular signaling occurs after EIII binds to the target cells, and whether this cellular response is associated with hemorrhage pathogenesis *in vivo* also remains unclear. Our previous study revealed that treatment with viral-load-equivalent levels of EIII in DHF can suppress megakaryopoiesis through autophagy impairment and cell death (7), indicating that the binding of EIII to endothelial cells may involve cytotoxicity. Concerning viremia exacerbation, circulating viral-particle surface-EIII is theoretically increased to cytotoxic levels in the plasma of patients with dengue during peak viremia. Consistent with this premise, vascular leakage follows peak viremia (5). Therefore, in the present study, we hypothesized that EIII would be a virulence factor leading to endothelial damage.

Because secondary DENV infection in DHF causes vascular damage and abnormal immune responses in addition to viremia-induced damage, abnormal immune responses against dengue are considered a pathogenic factor of DHF. DENV infection and immunization of viral protein NS1 lead to the production of autoantibodies (8–14), which could constitute an abnormal immune response. In our previous study, two sequential injections of DENV and antiplatelet immunoglobulin (Ig), which simulate peak viremia and abnormal immunity, respectively, successfully induced Nlrp3 inflammasome–mediated hemorrhage in mice (10). In the present study, we investigated whether EIII exposure would lead to endothelial dysfunction, whether sequential injections of EIII and antiplatelet Ig would elicit hemorrhage in mice, and whether such pathogeneses are Nlrp3 inflammasome–mediated responses. We also discuss relevant regulations and potential applications.

## Materials and Methods

### DENV, Recombinant Protein, Antibodies, and Analyses Kits

Soluble recombinant proteins DENV NS1 (rNS1), EIII (rEIII), and glutathione-S transferase (rGST) and DENV-2 (strain PL046) were obtained and purified as described previously (7, 10). To reduce endotoxin contamination (i.e., that of lipopolysaccharide [LPS]) to a desired concentration (<1 EU/mg of protein), the lysate- and resin-loaded column was washed with a buffer (8 M urea, 100 mM NaH_2_PO_4_, and 10 mM Tris-HCl; pH = 6.3) with the addition of 1% Triton X-114 (Sigma-Aldrich, St. Louis, MI, USA) (15). The rEIII was eluted with a buffer (8 M urea, 100 mM NaH_2_PO_4_, and 10 mM Tris-HCl; pH = 4.5) and refolded using a linear 0–4 M urea gradient in a dialysis buffer (2 mM reduced glutathione, 0.2 mM oxidized glutathione, 80 mM glycine, 1 mM EDTA, 50 mM Tris-HCl, 50 mM NaCl, and 0.1 mM phenylmethylsulfonyl fluoride) at 4°C for 2–3 h, as described previously (7). The purity of the rEIII protein was approximately 90%. LPS contamination was monitored using a Limulus Amoebocyte Lysate QCL-1000 kit (Lonza, Walkersville, MD, USA) (7, 16, 17). Batches of purified recombinant proteins with LPS concentrations of less than 1 EU/mg of protein were used. The pre-immune control Ig concentration, anti-NS1 Ig concentration, and anti-EIII Ig concentration of the experimental rabbits (New Zealand White; *Oryctolagus cuniculus*) were obtained before and after immunization with rNS1 and rEIII, which was performed as described previously (13). Approximately 6–10 days after the fifth immunization cycle, anti-NS1 serum was collected from the 50% of the rabbits with the most significant elevation of antiplatelet IgG. The anti-EIII serum was obtained after the third immunization cycle. The IgG fractions were obtained and purified using a protein A column attached to a peristaltic pump (Amersham Biosciences; flow rate of 0.5–1 ml/min). Subsequently, they were washed and eluted (10). An antiplatelet monoclonal antibody (rat anti-mouse integrin αIIb/CD41 Ig, clone MWReg30; BD Biosciences, San Jose, CA, USA) was used as a positive control Ig for thrombocytopenia induction in mice, which was performed as described previously (18). For competition of between rEIII-endothelial cell binding, following recombinant proteins were used, recombinant mouse P-selectin, E-selectin, dendritic cell-specific intercellular adhesion molecule-3-grabbing non-integrin (DC-SIGN; CD209), DC-SIGNR, C-type lectin domain family 5 member A (CLEC5A), CLEC2, glycoprotein Ib (GPIbα; CD42b) (R&D Systems, Indianapolis, IN, USA). To analyze the binding properties of rEIII proteins on endothelial HMEC-1 cells, DENV virus particles and rEIII protein were conjugated with biotin by using an EZ-Link™ Sulfo-NHS-Biotinylation kit (Thermo Fisher Scientific). The levels of biotin-labeled rEIII proteins bound to endothelial cells (50 µg/ml protein + (2 × 10^5^) cells/ml in culture medium for 30 min) were determined through flow cytometry by using PE/Cy5 avidin (BioLegend, San Diego, CA, USA) staining. The rEIII-competitive inhibitor chondroitin sulfate B (CSB, 10 µg/ml; Sigma-Aldrich, St. Louis, MO, USA) was used to suppress rEIII-induced endothelial cell binding and cell death. Anti-P-selectin Ig (50 µg/ml; BD Biosciences) and isotype control Ig (50 µg/ml; mouse IgG1, κ isotype control antibody, BioLegend) was used for stimulating HMEC-1 cell ROS and pyroptosis.

### Experimental Mice

Wild-type C57BL/6J mice aged 8–12 weeks were purchased from the National Laboratory Animal Center (Taipei, Taiwan) (14, 19–21). Genetically deficient mice with a C57BL/6J background, including *Nlrp3* ^−/−^ and *Casp1* ^−/−^ knockout (KO) mice, were obtained from the Centre National de Recherche Scientifique (Orléans, France) (10, 22). All animals were housed in the Animal Center of Tzu-Chi University in a specific-pathogen-free environment controlled for temperature and lighting, and were given free access to food and filtered water. All genetic knockout strains were backcrossed with the wild type for at least six generations.

### Ethics Statement

The animal experiments in this report were conducted in accordance with the national directive for the protection of laboratory animals (Taiwan Animal Protection Act, 2008). All experimental protocols related to animal use were approved by the Animal Care and Use Committee of Tzu-Chi University, Hualien, Taiwan (approval ID: 101019).

### Analysis of Blood Parameters and Liver Function

Blood samples were collected from the retro-orbital plexus of the mice using plain capillary tubes (Thermo Fisher Scientific, Waltham, MA, USA) and transferred into polypropylene tubes (Eppendorf; Thermo Fisher Scientific) containing an anticoagulant acid–citrate–dextrose (ACD) solution (38 mM citric acid, 75 mM sodium citrate, 100 mM dextrose) (23, 24). Platelet-poor plasma was prepared by centrifugation at 1,500 × g for 20 min. To remove contaminant cells, it was then centrifuged at 15,000 × g for 3 min. The platelet counts of mice were determined using a hematology analyzer (KX-21N; Sysmex, Kobe, Japan). Mouse liver function was analyzed through the detection of concentrations of an enzyme specifically expressed by liver cells, namely circulating aspartate transaminase (AST), by using a clinical biochemistry analysis system (COBAS INTEGRA800, Roche).

### Endothelial Cell Analysis

Human microvascular endothelial cells (HMEC-1; Centers for Disease Control and Prevention, Atlanta, GA) (12) were treated with vehicle (normal saline), rGST (25 µg/ml), rEIII (25 µg/ml), and DENV (5 × 10^4^ PFU/ml), with or without additional treatments of 10 µg/ml heparin (China Chemical and Pharmaceutical Co., Taipei, Taiwan), de-N-sulfated heparin (Sigma-Aldrich), and EIII-competitive inhibitor chondroitin sulfate B (CSB; Sigma-Aldrich), respectively. After various treatments, freshly collected conditioned medium and HMEC-1 cells were subjected to flow cytometry analysis of reactive oxygen species (ROS) using 2′,7′-dichlorofluorescin diacetate (Sigma-Aldrich) and caspase-1 activity (BioVision, Milpitas, CA, USA) or were stored at −80˚C before further cytokine and RNA experiments. Concentrations of proinflammatory cytokines (i.e., interleukin [IL]-1β, tumor necrosis factor [TNF]-α, and IL-6; BioLegend) and soluble thrombomodulin (Abcam, Cambridge, UK) were determined through enzyme-linked immunosorbent assay (ELISA). Levels of EIII-stimulated (0.6 µM, 24, 48, 72 h) HMEC-1 cell released soluble form thrombomodulin (sTM) and EIII-elicited (2 mg/Kg, 24 h) mouse circulating sTM were analyzed using a human and a mouse thrombomodulin ELISA Kit, respectively. Total cellular RNA was prepared using an RNeasy Mini Kit (Qiagen). RNA concentration was quantified through spectrophotometry at 260 nm (NanoDrop2000c; Thermo Fisher Scientific). By using the iScript cDNA Synthesis Kit (Bio-Rad, Foster City, CA, USA), 1 mg of total RNA was reverse transcribed into cDNA. Quantitative real-time reverse transcriptase polymerase chain reaction qRT-PCR was performed using Maxima SYBR Green/ROX qPCR Master Mix (Thermo Scientific) with primers of the target genes (Nlrp3, NF-κb, IL-1β, TNF-α, and thrombomodulin) and β-actin as the internal control (**Table S1**). The results were analyzed using QuantStudio 5 qPCR and Thermo Fisher Cloud systems (ThermoFisher Scientific), as described previously (25).

### Endothelial Cell Death and Mitochondrial Analyses

To analyze DENV- or rEIII-induced endothelial cell death, HMEC-1 cells (2 × 10^5^) were incubated with DENV or rEIII for 1 h and then subjected to flow cytometry analyses after washing with PBS. Various RCD responses, namely including apoptosis (CaspGLOWTM Red Active Caspase-3 Staining Kit, BioVision, Milpitas, CA, USA), autophagy (Cyto-ID™ Autophagy Detection Kit, Enzo Life Sciences, Farmingdale, NY, USA), ferroptosis (C11 BODIPY 581/591, Cayman Chemical, Ann Arbor, MI, USA), necroptosis (RIP3/B-2 Alexa Fluor 488, Santa Cruz Biotechnology, Santa Cruz, CA, USA), pyroptosis (Caspase-1 Assay, Green, ImmunoChemistry Technologies, MI, USA), and live/dead cell labeling (Zombie NIR™ Fixable Viability Kit, BioLegend), were analyzed using respective cell labeling reagents (30 min in PBS). Treatments (1 h) of cell death inducers were used as positive controls for various types of RCD (apoptosis: doxorubicin, 2.5 μg/ml, Nang Kuang Pharmaceutical, Taipei, Taiwan; autophagy: rapamycin, 250 nM, Sigma-Aldrich; ferroptosis: erastin, 10 μM, Cayman Chemical; necroptosis: tumor necrosis factor alpha [TNF-α], 2 ng/ml, BioLegend; pyroptosis: nigericin, 3.5 μM, ImmunoChemistry Technologies, Minnesota, USA; 30 min in PBS). Inhibitors were used to address the involvement of specific RCD pathways (apoptosis: Z-DEVD-FMK, 10 μM, R&D Systems; autophagy: chloroquine diphosphate, 60 μM, Sigma-Aldrich; ferroptosis: ferrostatin-1, 2.5 μM, Cayman Chemical; necroptosis: necrostatin-1, 50 μM, Cayman Chemical; pyroptosis: Z-WHED-FMK, 10 μM, R&D Systems; ROS: N-acetylcysteine (NAC), 1 mM; 30 min pretreatments before addition of DENV, rEIII, and cell-death inducers). To analyze the induction of mitochondrial superoxide, MitoSOX™ Red mitochondrial superoxide indicator was used (Thermo Fisher Scientific, Waltham, MA, USA; 30 min in PBS) (26). For caspase-1 activity, an additional colorimetric kit was used (BioVision, Milpitas, CA, USA). Cell-permeant 2’,7’-dichlorodihydrofluorescein diacetate (DCFDA, Abcam, Cambridge, UK) staining was used to determine cellular ROS levels (**Figure S1**). Cellular ROS/superoxide detection kits (Abcam) were used for inhibitor and competition experiments (**Figures S4, S8, S9**).

### Effects of Sequential Injection of DENV and rEIII + Anti-NS1 Ig on Induction of Hemorrhage in Mice

As described previously (10), the mice were first given a subcutaneous injection of DENV (3 × 10^5^ PFU/mouse; DHF viral load (27); or concentrations of rEIII equivalent to the DHF viral load (i.e., 2 mg/kg). This was followed by a subcutaneous injection of antiplatelet IgG using either (1) anti-CD41 Ig (0.2 mg/kg rat monoclonal MWReg30; Pharmingen), which is well established for immune thrombocytopenia (ITP) induction (18) or, 24 h later, (2) anti-NS1 Ig (8.5 mg/kg rabbit polyclonal, ITP-inducible) (13). Anesthesia was established 5 min before each injection (i.e., vehicle, rEIII, DENV, and Igs) through intraperitoneal injection of 2.5% avertin solution (in 10 ml/kg saline). We analyzed the platelet counts (Analyzer KX-21N; Sysmex) (18); hemorrhage classification (as described in the next section); concentrations of IL-1β, TNF-α, IL-6, and soluble thrombomodulin (ELISA, BioLegend and Abcam); and, using a chromogenic assay, the expression levels of the anticoagulant proteins antithrombin III and protein C (Sekisui Diagnostics) 24 h after the antiplatelet Ig injection. To measure the plasma leakage, a single intravenous injection of Evans blue dye along with the sequential Ig injections was administered (12). The mice were sacrificed 12 h after dye treatment, and their tissue was collected and minced. The dye retention rates were determined from a standard curve of Evans blue in formamide by using a spectrophotometer (Hitachi, Tokyo, Japan). Inhibitors and drugs, such as ROS scavenger N-acetyl-l-cysteine (NAC; Sigma-Aldrich, 50 mg/kg), CSB (Sigma-Aldrich, 0.5 mg/kg), caspase 1 inhibitor Z-WEHD-FMK (R&D Systems, 10 mg/kg), recombinant IL-1 receptor antagonist (IL-1RA) (PeproTech, 0.8 mg/kg), TNF-α inhibitor etanercept (Pfizer, 10 mg/kg), and IVIg (Bayer, 2 g/kg), were administered subcutaneously before (CSB and NAC: concurrent with rEIII injection; Z-WEHD-FMK, IL-1RA, etanercept: 10 min after rEIII injection) or after (IVIg: 10 min after Ig injection) the anti-CD41/anti-NS1 Ig injections.

### Hemorrhage Classification

We graded the severity of the hemorrhage induced by the sequential treatments of rEIII + anti-NS1 Ig and DENV + anti-NS1 Ig as described previously (10). Digitized images (RGB color mode, 0.75 × 0.6 cm^2^, 600 dpi) of the hemorrhagic lesions in the mice were captured under standard conditions: the illumination density was 200 lx, the lighting element was a 20 W Philips fluorescent lamp, a Canon IXUS-860IS camera was used, and the sample-to-camera distance was 7 cm. Adobe Photoshop software was then used to obtain the red and green signals without additional adjustment of brightness or contrast. The red and green intensities in a particular image were measured using ImageJ software (v1.46, NIH). Approximate values of hemorrhage score were calculated by subtracting the image intensity of the red signals from that of the green signals (10).

### Statistical Analysis

The means, standard deviations, and other summary statistics of the quantifiable data were calculated using SigmaPlot 10 and SPSS 17 software. The data were subjected to one-way analysis of variance (ANOVA), followed by the post-hoc Bonferroni-corrected *t* test. A probability of type 1 error (α) of.05 was used to determine statistical significance.

## Results

### rEIII-Induced ROS Stress, Proinflammatory Phenotype, and Cell Death in Endothelial Cells

**Figure 1:**
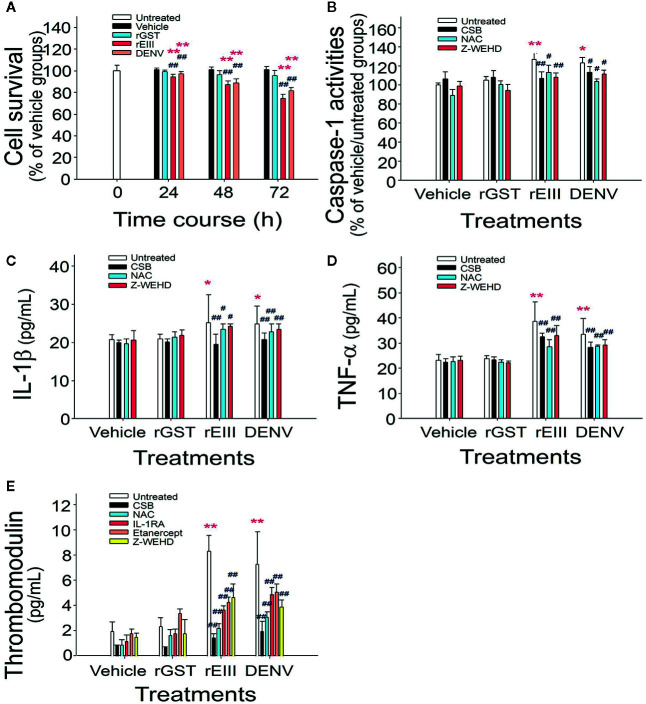
Endothelial inflammatory responses induced by rEIII *in vitro*. Treatments of recombinant rGST, rEIII, and DENV on endothelial HMEC-1 cell defects at various time courses were analyzed **(A)**. After rGST, rEIII, and DENV treatments, with or without the addition of inhibitors, the levels of caspase-1 activity **(B)**, IL-1β release **(C)**, TNF-α release **(D)**, and soluble thrombomodulin release **(E)** were analyzed—specifically, 24 h **(B–D)**, and 48 h **(E)** after the treatments. Glycosaminoglycans for rEIII binding competition: CSB. Inhibitors: NAC for ROS; Z-WEHD-FMK (Z-WEHD) for caspase-1 and IL-1; IL-1RA for IL-1; etanercept for TNF-α. ANOVA: *p < 0.05 and **p < 0.01 vs. the vehicle groups; ^#^ *p* < 0.05 and ^##^ *p* < 0.01 vs. groups not treated with inhibitors (*n* = 6; three experiments with two replicates).

Studies have demonstrated that DENV binds to endothelial cell glycoproteins through envelope proteins. However, it is unclear whether such binding induces adverse effects similar to those previously observed in megakaryocytes (7). In analyses of proinflammatory and proapoptotic responses and soluble thrombomodulin release, which is a marker of endothelial damage (28), we observed that treatment with levels of rEIII and DENV equivalent to the DHF viral load induced death of HMEC-1 cells. The rEIII-induced endothelial cytotoxicity was associated with increased levels of caspase-1 activity, as well as with increased concentrations of IL-1β and soluble thrombomodulin (**Figure 1** and **Figure S1**). Consistent with these findings, qRT-PCR revealed that the mRNA expression of NF-κB, IL-1β, TNF-α, inflammasome component Nlrp3, and thrombomodulin increased notably after treatment with rEIII and DENV (**Figure S2**). Treatments with CSB, NAC, and Z-WEHD-FMK substantially reduced the release of IL-1β, TNF-α, and thrombomodulin (**Figure 1**). These results suggest that the DENV-induced proinflammatory phenotype of endothelial cells is likely mediated through EIII, as well as through the induction of downstream ROS production and caspase-1 activity.

### Pyroptosis Is a Major Regulated Cell Death Pathway in Endothelial Cells Treated With DENV and rEIII

**Figure 2:**
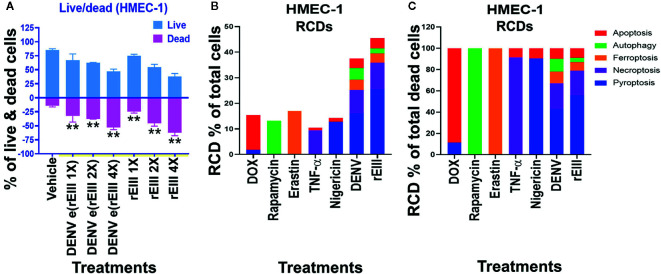
DENV- and rEIII-induced RCD in endothelial cells. **(A)** HMEC-1 endothelial cells treated with vehicle and various doses of DENV and rEIII; the live or dead status of the cell populations were determined using Zombie-NIR Kit labeling and flow cytometry analysis. [rEIII 1× = 0.3 µM, 2× = 0.6 µM, 4× = 1.2 µM; DENV e(rEIII 1×) is a DENV level equivalent to 0.3 µM rEIII]. **(B)** RCD inducers, doxorubicin (DOX; apoptosis) (2.5 µg/ml), rapamycin (autophagy) (0.5 µM), erastin (ferroptosis) (10 µM), TNF-α (necroptosis) (2.5 ng/ml), and nigericin (pyroptosis) (3.5 µM) induced relatively simple RCD patterns (flow cytometry gating and calculation methods described in **Figure S3**). By contrast, DENV and rEIII induced multiple RCD pathways, in which pyroptosis was the main RCD response, accounting for approximately 50% of total RCD response. If we normalize the respective RCD by the population of death cells (dead cell population normalized to 100%), we can obtain a more similar RCD pattern in DENV and rEIII groups **(C)**. **P < 0.01 vs. vehicle groups.

**Figure 3:**
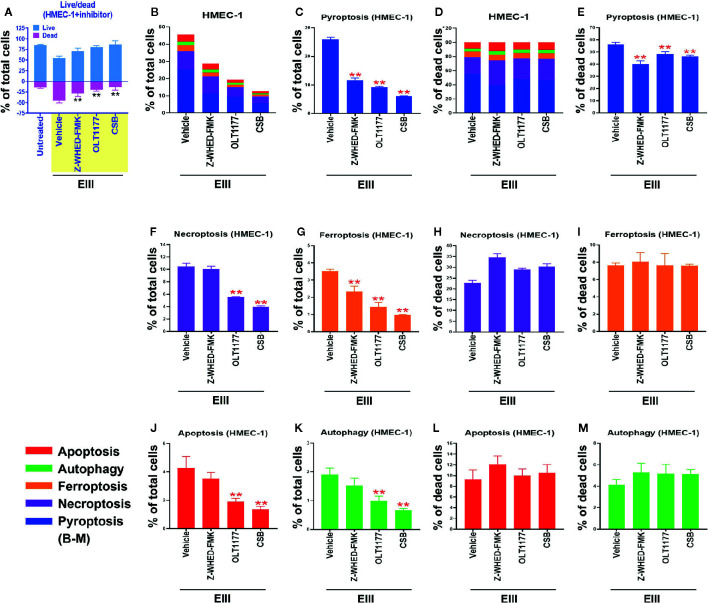
Protection of endothelial cells from rEIII-induced pyroptosis under treatment with Nlrp3 inflammasome inhibitors. Treatment with Nlrp3 inhibitor CSB (chondroitin sulfate B, 10 μg/ml), OLT1177 (10 µM) and caspase 1 inhibitor Z-WHED-FMK (10 µM) rescued rEIII-induced endothelial cell death **(A)**. Treatments with CSB, OLT1177 and Z-WHED-FMK rescued cell dead population (**B, C, F, G, J, K**; cells with RCDs). If we normalize the respective RCD % by the population of death cells (**D**: dead cell population normalized to 100%), we found that CSB, OLT1177 and Z-WHED-FMK preferentially rescued pyroptosis **(E)**, but not the other tested RCDs **(H, I, L, M)**. n = 6, **P < 0.01, significant suppression vs. vehicle groups.

Results from annexin V staining, terminal deoxynucleotidyl transferase dUTP nick end labeling (TUNEL), propidine iodide (PI) staining, and caspase assays suggested the involvement of apoptosis in DENV-induced endothelial cell death (29, 30). However, because annexin V, TUNEL, PI, and caspase signals can all be detected in nonapoptotic cell death (31–35), we investigated whether pathways of nonapoptotic regulated cell death (RCD) were also involved in DENV- and rEIII-induced endothelial cell death. Specifically, endothelial RCD pathways, including pyroptosis, necroptosis, ferroptosis, apoptosis, and autophagy, were analyzed. In line with the data on IL-1β, TNF-α, and thrombomodulin (**Figure 1** and **Figures S1–S3**), DENV and rEIII treatment induced endothelial cell death in a dose-dependent manner (**Figure 2A**). Overall, various cell death inducers, including doxorubicin (apoptosis) (36, 37), rapamycin (autophagy) (38), erastin (ferroptosis) (39), TNF-α (necroptosis) (40, 41), nigericin (pyroptosis) (42), served as positive control agents to induce respective cell death pathway of the tested endothelial cells (**Figures 2B, C**, dead cell population adjusted to 100%; **Figure S3**, flow cytometry gating and calculation). Notably, when compared with cell death agonists, DENV and rEIII treatment induced considerable pyroptosis, necroptosis, and ferroptosis responses in the endothelial cells, but only minor manifestations of apoptosis (**Figure 2B**, % of total cells; 2C, % of total dead cell). In addition, the cell type specific RCD patterns (CTS-RCDPs) in the DENV- and rEIII-treated groups were somewhat similar, with pyroptosis exhibiting the highest levels in both groups among all tested RCD pathways (**Figure 2B**; approximately 50%), suggesting that DENV-induced CTS-RCDP in the endothelial cells is likely mediated through EIII on the DENV virion. Accordingly, whether the Nlrp3 inflammasome was involved in such pyroptosis responses was determined through treatments with Nlrp3 inhibitor OLT1177 and inflammasome/caspase-1 inhibitor Z-WHED-FMK, which resulted in substantial mitigation of endothelial death (**Figure 3A**) and pyroptosis (**Figures 3B–E**). The treatments also suppressed the relatively minor manifestations of necroptosis, ferroptosis, apoptosis, and autophagy in the endothelial cells (**Figures 3F, G, J, K**). Intriguingly, when the RCD % normalized with total dead cell population (D: dead cell %, normalized to 100%), we found that both inhibitors OLT1177 and Z-WHED-FMK preferentially suppressed pyroptosis (**Figure 3E**); while, no suppression on necroptosis (**Figure 3H**), ferroptotic (**Figure 3I**), apoptotic (**Figure 3L**) and autophagic (**Figure 3M**) was observed. Taken together, the results indicate that pyroptosis is the major RCD of rEIII-induced endothelial cell death, and that these cells can be rescued from apoptosis by selective inhibitors against the Nlrp3 inflammasome.

### Treatment With Nlrp3 Inflammasome Inhibitor OLT1177 Ameliorates DENV- and rEIII-Induced Endothelial Cell Pyroptosis and the Metabolic Burden on Mitochondria

**Figure 4:**
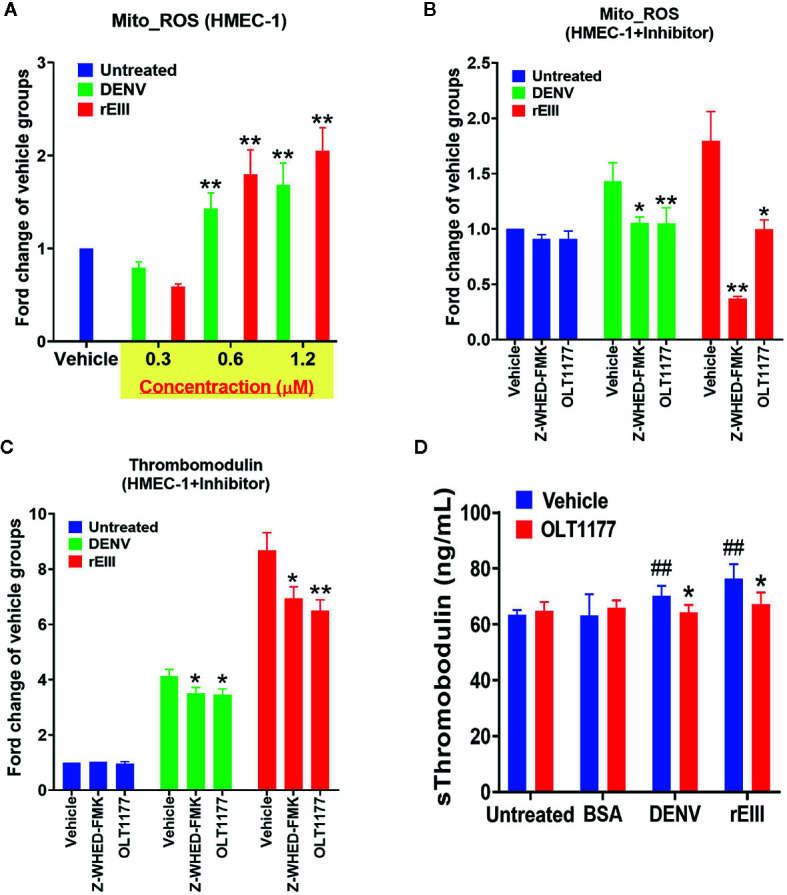
Protection of endothelial cells from DENV- and rEIII-induced damage and metabolic burden under treatment with Nlrp3 and caspase-1 inhibitors. Dose-dependent elevation of mitochondria superoxide levels under DENV and rEIII treatment **(A)**. Suppression of mitochondria superoxide levels and induction of surface thrombomodulin expression under treatment with Nlrp3 and caspase 1 inhibitors Z-WHED-FMK and OLT1177, respectively **(B, C)**. Amelioration of DENV- and rEIII-induced thrombocytopenia in C57BL/6J mice under OLT1177 treatment **(D)**. *n* = 6, and ^##^ *p* < 0.01 vs. the untreated groups; *p < 0.05 and **p < 0.01 vs. the vehicle groups.

Because inflammasome-mediated pyroptosis was determined to be major RCD involved in DENV- and rEIII-induced endothelial dysfunction, we further explored whether suppression of the Nlrp3 inflammasome through inhibition would attenuate this dysfunction. As shown in **Figure 4A**, DENV and rEIII treatment increased mitochondria superoxide levels in a dose-dependent manner. As shown in **Figure 4B**, Nlrp3 inflammasome inhibitors OLT1177 and Z-WHED-FMK alleviated such metabolic burdens on endothelial mitochondria, as well as inducing and releasing thrombomodulin, a marker of endothelial damage (**Figure 4C**). In line with these results, OLT1177 treatment resulted in the mitigation of DENV- and rEIII-induced elevation of circulating soluble thrombomodulin (sTM) in the mice (**Figure 4D**). To clarify whether ROS and Nlrp3 inflammasome activate each other, antioxidant NAC and inflammasome inhibitors OLT1177 and Z-WHED-FMK were used. Here we found that all 3 inhibitors, NAC, OLT1177 and Z-WHED-FMK, suppressed EIII-induced ROS and caspase-1 activations (**Figure S4**); the results suggest a positive feedback regulation exists between ROS and caspase-1. These results collectively suggest that EIII is a virulence factor in the induction of endothelial defects, and that the Nlrp3 inflammasome is a critical target for DENV and EIII to induce endothelial dysfunction.

### Treatment With rEIII Served as a First Hit for Hemorrhage Induction in a Two-Hit rEIII + Autoantibody Mouse Model

**Figure 5:**
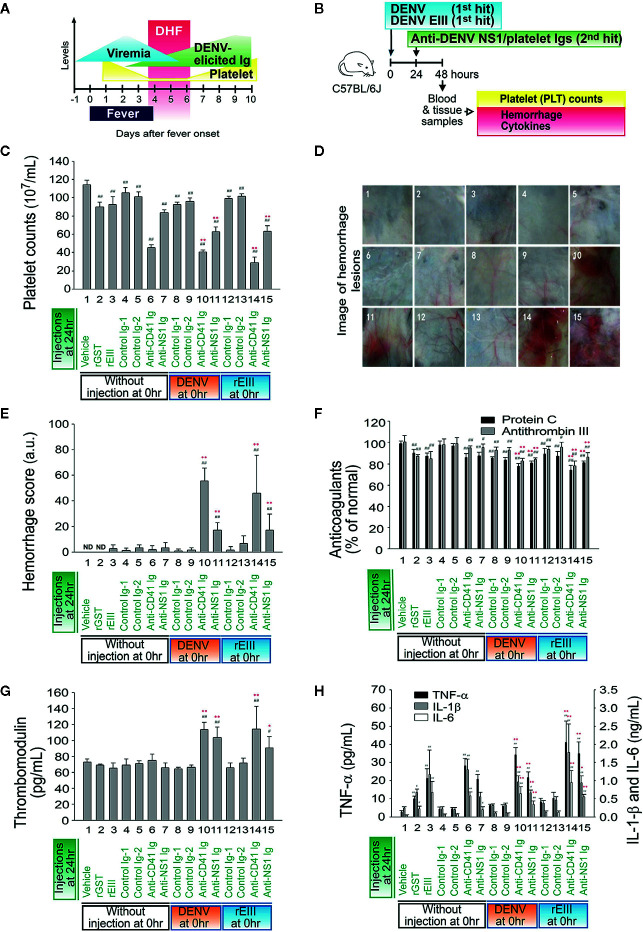
Comparisons between the induction of pathophysiology under treatment with rEIII and DENV. Time course of clinical parameters during DHF **(A)** and the experimental outline **(B)** are shown. **(C–H)** Mice treated with DENV (panels 8–11; 3 × 10^5^ PFU/mouse; first hit), rEIII (panels 12–15; 2 mg/kg equivalent to 3 × 10^5^ PFU/mouse; first hit) or mice not treated with first hit injection (panels 1–7; vehicle groups) underwent autoantibody treatment (second hit). Parameter changes in **(C)** platelet counts, **(D)** hemorrhagic lesions, **(E)** hemorrhage score, **(F)** anticoagulant protein C and antithrombin III, **(G)** soluble thrombomodulin, and **(H)** proinflammatory cytokines TNF-α, IL-1β, and IL-6 were then recorded. Data are presented as means ± standard deviations. *p < 0.05 and **p < 0.01 indicate significantly worse conditions vs. DENV + control Ig groups; ^#^ *p* < 0.05 and ^##^ *p* < 0.01 vs. the vehicle groups. *n* = 6 (three independent experiments with two replicates). The mouse drawing used in this and following figures was originally published in the Blood journal: Huang, **(H)** S., D-SS, T-SL, and H-HC. Dendritic cells modulate platelet activity in IVIg-mediated amelioration of ITP in mice. Blood, 2010; 116: 5002–5009. ^©^ the American Society of Hematology.

The rEIII injection induced elevation of circulating sTM in the mice (**Figure 4D**), a sign of endothelial damage; however, they did not develop symptoms of hemorrhage. As mentioned, the sequential injection of DENV and anti-NS1 Ig in a two-hit model induced hemorrhage in mice in our previous study (10). In the present study, DHF-viral-load-equivalent levels of rEIII induced endothelial cell defects (**Figures 1** **–** **4**). We further investigated whether rEIII treatment may serve as the first hit for hemorrhage induction. Notably, compared with the rEIII and rEIII + control Ig treatments, the rEIII + anti-NS1 Ig treatment induced more considerable vascular leakage in mouse tissue, including that of the lung, liver, ileum, and skin (**Figure S5**, Evans blue data). This is partly consistent with the tissue injury observed in patients with DHF (2). We then used thrombocytopenia, plasma leakage (indicated as hypoproteinemia), and high levels of circulating AST, which are acknowledged by the World Health Organization (WHO) as standard parameters in DHF assessment (43, 44), to evaluate pathogenic alterations in mice. Sequential injection of rEIII + anti-NS1 Igs or rEIII + anti-CD41 Igs (CD41 is a putative anti-NS1 Ig targets on platelets) (45) significantly exacerbated thrombocytopenia, plasma leakage, and liver damage (**Figure S6**). Compared with the mice in the single-injection groups, mice that received combined treatments of DENV and rEIII + anti-NS1 Igs or DENV and rEIII + anti-CD41 Igs but not control Igs (i.e., pre-immune and isotype control Igs) developed significant thrombocytopenic responses (**Figure 5A**, DHF clinical course; **Figure 5B**, experiment outline; **Figure 5C**, platelet counts) and exhibited greater hemorrhage severity (**Figures 5D, E**); anticoagulant suppression (**Figure 5F**); endothelial cell damage (**Figure 5G**), and higher expression of IL-1β, TNF-α, and IL-6 (**Figure 5H**). These results collectively suggest that with rEIII as a first hit, the pathophysiological changes observed in the two-hit mouse model of DENV and rEIII + autoantibody are similar to those detected in patients with DHF (43, 44).

### Involvement of the Nlrp3 Inflammasome in the rEIII + Autoantibody Two-Hit Model

**Figure 6:**
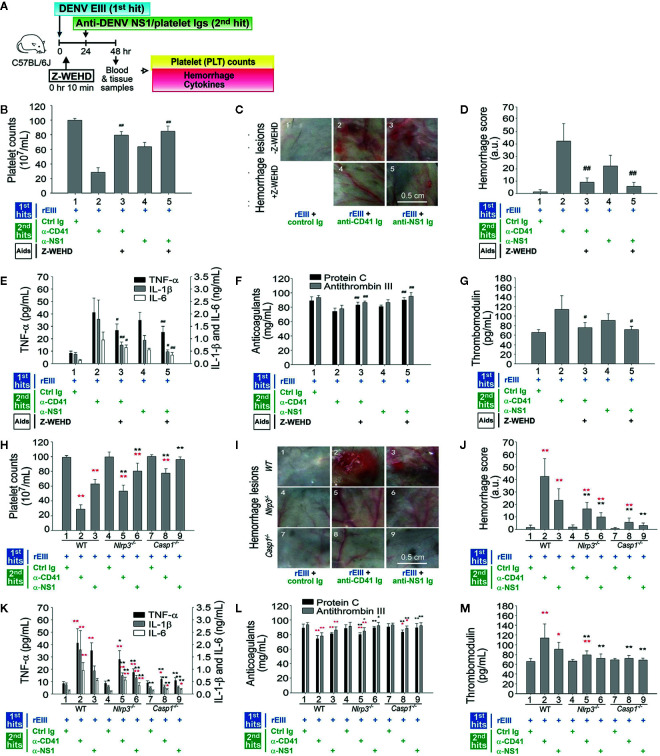
Involvement of the Nlrp3 inflammasome pathway in pathophysiological changes induced in the two-hit rEIII + autoantibody model. Experimental outline **(A)** and the pathophysiological changes in mouse platelet counts **(B, H)**, hemorrhagic lesions **(C, D, I–J)**, proinflammatory cytokines TNF-α, IL-1β, and IL-6 **(E–K)**, anticoagulant protein C and antithrombin III **(F, L)**, and soluble thrombomodulin **(G, M)** are shown. **(B–G)** Treatment with selective caspase-1 inhibitor Z-WEHD-FMK reduced hemorrhage and inflammation of wild-type mice in a two-hit rEIII + anti-NS1 Ig model (panels 3 and 5 vs. 2 and 4, respectively). **(H–M)** Hemorrhagic and inflammatory manifestations after rEIII and antibody treatments (*n* = 6) in the *Nlrp3* ^−/−^ and *Casp1* ^−/−^ mice (panels 4–6 and 7–9, respectively) were compared with those in wild-type mice (panels 1–3). Data are presented as means ± standard deviations. *p < 0.05 and **p < 0.01 indicate significantly worse conditions vs. those in the rEIII + control Ig groups of the respective strains; ^#^ *p* < 0.05, ^##^ *p* < 0.01, *p < 0.05, and **p < 0.01 indicate significant mitigation of pathophysiological presentations compared with those in the rEIII + anti-CD41 (α-CD41)/anti-NS1 (α-NS1) Ig groups of the respective strains. The mouse drawing in this figure was originally published in Huang et al. (18).

The involvement of the Nlrp3 inflammasome in DENV infection was revealed in the *in vitro* experiments (**Figures 1** **–** **4**) and in the two-hit mouse model of DENV + autoantibody (10). Evaluations were further conducted to determine whether the Nlrp3 pathway was involved in pathogenesis *in vivo* mediated by the rEIII + autoantibody two-hit model. Treatment with Z-WEHD-FMK notably mitigated all the pathological changes induced by the two-hit protocol, suggesting that the caspase-1 pathway plays a role in the pathogenesis (**Figure 6A** experiment outline, **Figures 6B–G**). In addition, a two-hit rEIII + autoantibody treatment was administered to *Nlrp3* ^−/−^ and *Casp1* ^−/−^ mice. All the mentioned pathological changes were substantially more reduced in both types of mice than in the wild-type controls (**Figures 6H–M**). These results suggest that the Nlrp3 inflammasome is involved in pathogenesis mediated by the two-hit rEIII + autoantibody protocol.

### Mitigation or Reversal of Pathophysiological Changes Mediated by the Two-Hit rEIII + Autoantibody Model Using IL-1 and TNF-α Blockers

**Figure 7:**
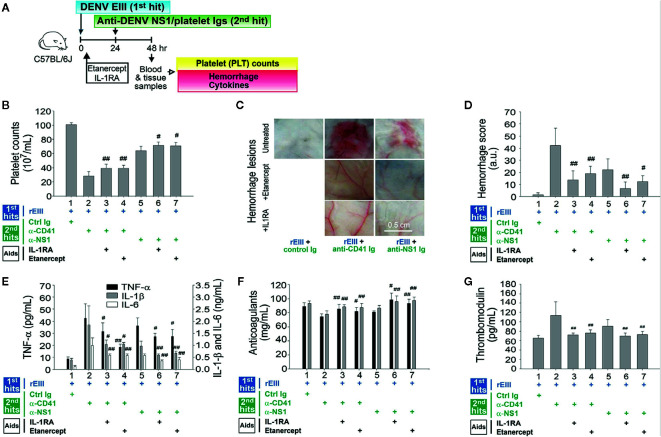
Involvement of TNF-α and IL-1β pathways in pathophysiological changes in a two-hit rEIII + autoantibody model. Experimental outline **(A)** and changes in mouse platelet counts **(B)**, severity of hemorrhagic lesions **(C–D)**, expression of proinflammatory cytokines TNF-α, IL-1β, and IL-6 **(E)**, anticoagulant protein C and antithrombin III **(F)**, and soluble thrombomodulin **(G)** are shown. Treatments with IL-1 inhibitor IL-1RA and TNF-α inhibitor etanercept **(B–G)** reduced hemorrhage and inflammation in the wild-type mice (*n* = 6). Data are presented as means ± standard deviations. ^#^ *p* < 0.05 and ^##^ *p* < 0.01 indicate significant mitigation of pathophysiological presentations compared with that in the EIII + α-CD41 and EIII + α-NS1 groups. The mouse drawing in this figure was originally published in Huang et al. (18).

IL-1β release was found to depend on inflammasome activation. IL-1β and TNF-α pathways were involved in endothelial cell defects that were induced by rEIII treatment alone (**Figure 1**). IL-1β and TNF-α blockers attenuated endothelial cell damage under treatment with rEIII only (**Figure 1**) and in the DENV + autoantibody two-hit mouse model (10). However, whether cytokine inhibitors can be used to mitigate or reverse the pathophysiological changes mediated by the two-hit rEIII + autoantibody model warrants further investigation. In the present study, treatment with IL-1RA (anti-IL-1) and etanercept (anti-TNF-α) resulted in substantial reductions of all pathophysiological changes mediated by the two-hit rEIII + anti-NS1 Ig model (**Figure 7**). These results suggest that the pathophysiological changes mediated by the two-hit rEIII + anti-NS1 Ig model can be mitigated through the inhibition of pathways involving IL-1 and TNF-α.

## Discussion

As a glycosaminoglycan (GAG) binding lectin (carbohydrate-binding protein) (46) and a cell-receptor-binding domain (6), EIII folds independently of other E subdomains while retaining its structure (47). Because EIII is exposed and accessible on virion surfaces (48), treatments with soluble EIII or EIII-neutralizing antibodies inhibit viral infection (47). Therefore, EIII is an attractive target for developing vaccines and antiviral agents against DENV (47). Beyond their effects against DENV infection and replication, rEIII treatments effectively and directly suppress megakaryocyte function (7). In the present study, we consistently found that endothelial cells were damaged by rEIII treatment (**Figures 1** **–** **4**). In fact, numerous other well-characterized lectins, such as plant lectins concanavalin A (Con A), mistletoe lectin (ML), and polygonatum cyrtonema lectin (PCL) exhibit similar effects against cell damage (49). When cells are treated with these plant lectins, ROS production is crucial to apoptotic induction (50–52). Moreover, the TNF-α pathway plays a critical role in lectin-induced cytotoxicity. For example, the cell-damaging effect of Con A is substantially reduced when the target cells do not express the TNF receptor (53, 54). Studies have indicated that ML treatment induces TNF-α expression (55), and that PCL treatment enhances TNF-α-induced apoptosis (56). Similarly, in the present study, we found that rEIII treatment induced endothelial ROS production, TNF-α and IL-1β release, and caspase-1 activation, all contributing responses to endothelial cell death (**Figure 1** and **Figure S1**). Treatments with inhibitors against the Nlrp3 inflammasome considerably attenuated rEIII-induced endothelial cell death *in vitro* and notably reduced hemorrhage in the two-hit rEIII + autoantibody model (**Figures 1** **–** **4**, **6** **,** **7**).

Cellular targets of the aforementioned lectins are carbohydrate moieties of cell surface glycosylated molecules, and cellular signaling overload constitutes a potential mechanism of cell damage induction (49). Because of its high capacity to induce cell death, lectin treatment has been proposed as an anticancer therapy (49, 57, 58). Although both plant- and animal-origin lectins have been investigated (49, 57, 58), the cytotoxic properties of viral lectins have not been extensively studied. In addition to DENV-EIII (7), Langat flavivirus envelope protein has been found to exhibit proapoptotic effect (59). However, whether such viral-protein-induced cytotoxicity contributes to viral pathogenesis *in vivo* remains to be determined.

As a GAG binding lectin (60), EIII could have multiple endothelial cell surface targets. Recent evidences have suggested that lectin DC-SIGN mediated DENV infection in dendritic cells (61); lectin CLEC2 mediates DENV-induced inflammation (62); and glycoprotein Ib (GP1bα; CD42b) is involved in DENV infection (63). CLEC2, CD42b, and the DC-SIGN isoform DC-SIGNR are expressed by some endothelial cell subpopulations (64–66). Here we used recombinant soluble DC-SIGNR, DC-SIGN, CLEC2, CLEC5A, CD42b, plus two additional endothelial lectins [P-selectin (**Figure S7**, HMEC-1 expression), E-selectin] as controls, to perform EIII competition experiments. Analysis results revealed that DC-SIGNR, P-selectin and E-selectin, but not CD42b or CLEC2 can block rEIII-endothelial cell binding (**Figure S8**). However, data further revealed that P-selectin displayed a markedly higher performance on the suppression of rEIII- endothelial cell binding, rEIII-induced cellular ROS up-regulation, endothelial cell death among all tested proteins (**Figure S8**). In agreement with DENV and rEIII treatments, anti-P-selectin antibody treatments also induced endothelial cell pyroptosis (**Figure S8D**). Furthermore, EIII treatments induced circulating thrombomodulin levels in wild type mice but not in P-selectin knockout mice (**Figure S8E**). These evidences collectively suggested that P-selectin is one of the cellular targets of EIII on endothelial cells. P-selectin is a cellular signaling receptor, with increased phosphorylation levels of cytoplasmic tail upon activation (67). P-selectin ligation by anti-P-selectin antibody known to induce platelet activation and enhances microaggregates (68). P-selectin is up-regulated on endothelial cell surfaces upon infection and inflammatory stimulations; and soluble P-selectin in turn exerts anti-inflammatory effects (23, 24, 69–71). As a critical inflammatory regulator, the role of P-selectin in DENV-mediated pathogenesis and is worthy of further investigations.

Cell population is heterogeneous even in one cell line. This could be a reason that reports have revealed treatments of pathogens and cytotoxic agents leading to multiple types of RCDs simultaneously (72–78). For example, when cellular stress can activate both receptor-induced lysosomal-dependent, and mitochondrial-mediated cell death pathways, which will lead to both programmed necrosis and apoptosis (74). Similarly, as DENV and EIII been reported to have multiple cellular targets, it is reasonable to detect multiple RCDs after DENV and EIII challenges. Here, we found that DENV and EIII but not the other cell death inducers, induced a similar RCD pattern in endothelial cells (**Figures 2B, C**). Although further investigations are needed, these endothelial CTS-RCDPs may be also useful on the characterization of specific pathway inhibitors; as inflammasome inhibitors OLT1177 and Z-WHED-FMK preferentially blocked pyroptosis, but not ferroptosis, apoptosis and autophagy (**Figure 3E** vs. **Figures 3I, L, M**).

In addition to EIII, NS1 was demonstrated to enhance endothelial permeability and vascular leaks through a toll-like receptor 4 (79, 80). Both DENV virus particle-associated EIII and soluble NS1 could be detected at high levels prior to the acute phase of DHF (81, 82), and may be considered as two virulence factors. Using a similar approach, we analyzed the induction of HMEC-1 cell pyroptosis after EIII and NS1 treatments (**Figure S9**). We found that both EIII and NS1 treatments can induce increased ROS and pyroptosis levels in endothelial cells, and EIII has a relative higher activity (**Figure S9**). Because the induction of virion-associated EIII and soluble NS1 are induced in a similar, but not a same time course (82), the respective pathogenic role of EIII and NS1 on the elicitation of DHF-related pathogenesis remains to be further studied. However, data obtained in the study suggested that virion-associated EIII is a candidate virulence factor that contributes to dengue-elicited endothelial cell injury.

In the present study, on the basis of the clinical course of DHF, in which viremia occurs before autoantibody elicitation, a proof-of-concept two-hit mouse model was designed, and hemorrhage was induced through sequential injection of DHF-viral-load-equivalent levels of DENV and an autoantibody in mice (10). Because DENV treatment can induce various pathogenic responses, further characterization was required to determine the major viral factors that contributed to the pathophysiological changes under the first hit. Although DENV infection processes have been studied extensively, the specific influence of cell–DENV binding on the development of dengue pathogenesis, especially in scenarios involving peak viral load, remains unclear. As mentioned, exposure to DHF-viral-load-equivalent levels of rEIII is sufficient for inhibiting autophagy and inducing apoptosis in megakaryocytes (7). In addition, treatments involving various plant and animal lectins can lead to cell damage (49). Hence, in the present study, we investigated the potential pathogenic effect of rEIII in a two-hit model. The rEIII treatment effectively induced hemorrhage in the two-hit rEIII + autoantibody mouse model. The Nlrp3 inflammasome, IL-1β, and TNF-α contributed to inflammation, coagulation defects, and hemorrhage.

The reasons for peak DHF manifestations being delayed until defervescence rather than accompanying peak viremia in the early stage of the disease have yet to be identified (81). Contrary to the results from various *in vitro* analyses, the primary infection targets of DENV in humans are probably dendritic cells rather than endothelial cells (83). Because the viremia titer is reduced substantially during defervescence, virion and DENV-infected cells are eliminated (44, 81). Therefore, the viral factors that contribute to vascular damage in DHF and the routes through which DENV causes vascular damage and plasma leakage during defervescence are worthy of investigation. Our results indicate that the binding of EIII (and probably virion-associated EIII in clinical cases) sufficiently results in endothelial cell damage (**Figure 1**). As shown in **Figure 5**, in the two-hit model, such damage led to hemorrhage only in instances of subsequent encounters with DENV-elicited autoantibodies. Levels of DENV-elicited antibodies increase considerably in the defervescence phase (44, 81); thus, the two-hit model may provide a reasonable explanation of DENV-induced vascular damage and plasma leakage during defervescence.

DHF is a life-threatening disease. No specific treatments against DENV infection are available, and unfortunately, some vaccine candidates have been determined to be not safe or ineffective (84, 85). The two-hit model described in the present study appears to be useful for delineating the mechanism of DENV-induced hemorrhage and for the development of a rescue strategy. Through a reductionist approach, we observed that rEIII treatment served as a first hit in hemorrhage induction in the two-hit mouse model. Treatment with the EIII-competitive inhibitor CSB considerably reduced rEIII-induced endothelial cell damage *in vitro*; moreover, in the mice, it reduced the hemorrhage induced by the rEIII + autoantibody sequential injection. In addition, the Nlrp3 inflammasome and RCD pathways involving IL-1β and TNF-α were involved in EIII-induced pathogenesis. The present results strongly indicate that the DENV-EIII virulence factor contributes to pathogenesis in DHF. The present findings constitute a valuable reference for the development of therapeutic strategies for managing DENV-induced hemorrhage in DHF.

## Data Availability Statement

The original contributions presented in the study are included in the article/**Supplementary Material** Further inquiries can be directed to the corresponding author.

## Ethics Statement

The animal study was reviewed and approved by Animal Care and Use Committee of Tzu-Chi University, Hualien, Taiwan (approval ID: 101019).

## Author Contributions

H-HC conceptualized and supervised this project. T-SL, D-SS, and C-YW performed experiments and analyzed the data. H-HC wrote this manuscript. All authors contributed to the article and approved the submitted version.

## Funding

Ministry of Science and Technology, Taiwan (101-2320-B-320-004-MY3, 105-2923-B-320-001-MY3, 107-2311-B-320-002-MY3), Tzu-Chi University (TCIRP95002; TCIRP98001; TCIRP101001), and Tzu-Chi Medical Foundation (TC-NHRI105-02; TCMMP104-06; TCMMP108-04; TCAS-108-01).

## Conflict of Interest

The authors declare that the research was conducted in the absence of any commercial or financial relationships that could be construed as a potential conflict of interest.
