# Supplementary material for: Exposure to Dengue Envelope Protein Domain III Induces Nlrp3 Inflammasome-Dependent Endothelial Dysfunction and Hemorrhage in Mice
Source: Front Immunol. 2021 Feb 25;12:617251. doi: 10.3389/fimmu.2021.617251 (PMC7947687; doi:10.3389/fimmu.2021.617251)
Supplement: Supplementary file 1 [file DataSheet_1.docx]

Supplementary Material

**Exposure to dengue envelope protein domain III induces Nlrp3 inflammasome-dependent endothelial dysfunction and hemorrhage in mice**

Te-Sheng Lien^1†^, Der-Shan Sun^1†^, Cheng-Yeu Wu ^2^, and Hsin-Hou Chang^1 *^

^1^ Department of Molecular Biology and Human Genetics, Tzu-Chi University, Hualien 970, Taiwan.

^2^  Center for Molecular and Clinical Immunology, Chang Gung University, Gueishan, Taoyuan 333, Taiwan.

† These authors share equals contribution

*** Correspondence:**Hsin-Hou Chang
hhchang@mail.tcu.edu.tw

Keywords: dengue hemorrhagic fever; dengue envelope protein domain III; two-hit dengue mouse model; endothelial damage; Nlrp3 inflammasome; pyroptosis; necroptosis; apoptosis

# Supplementary Figures 1-9

**Supplementary Table 1**

**Fig. S1**


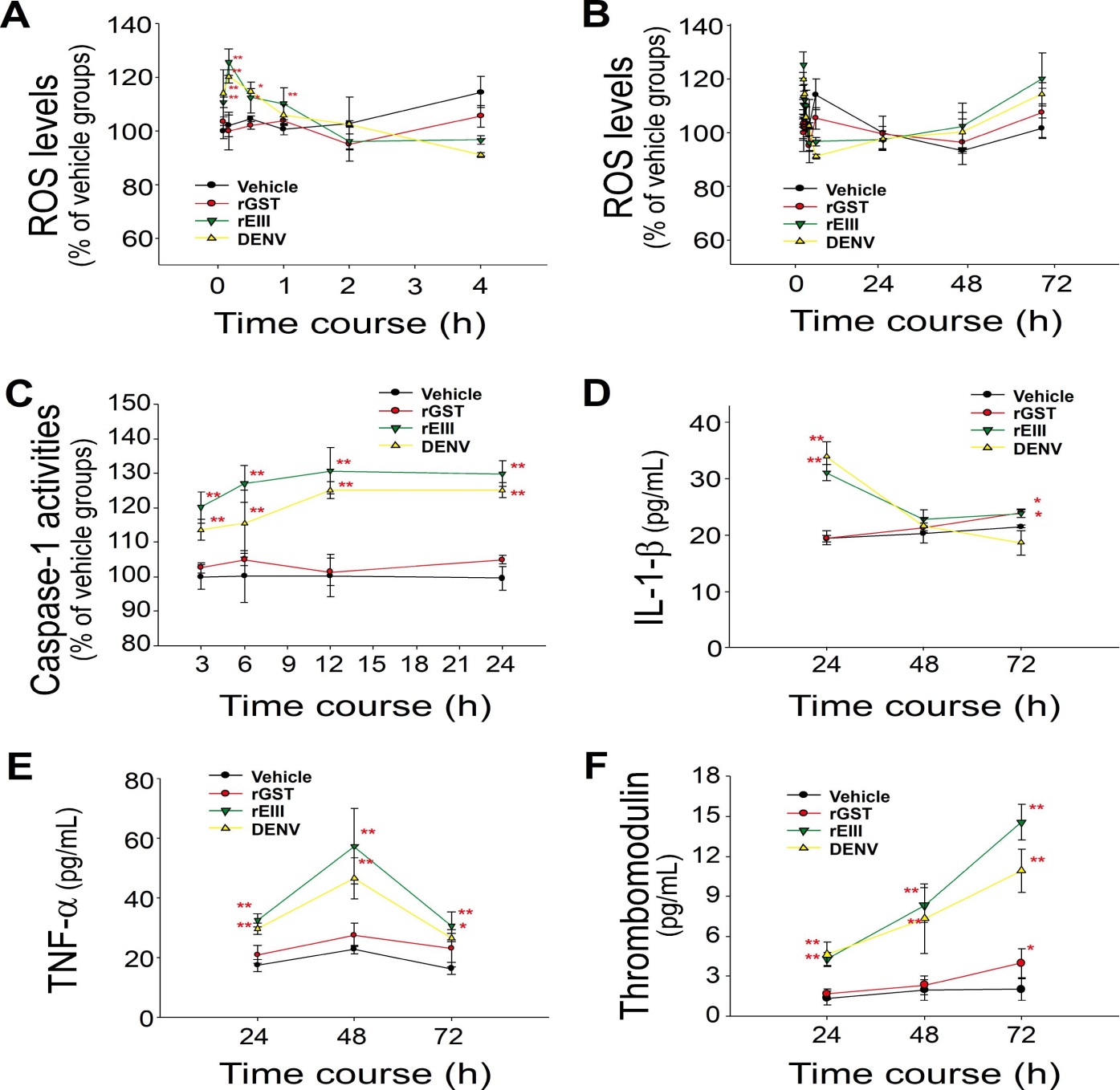


**Figure S1.** **Time course of rEIII-induced abnormal endothelial cell responses.** Treatments of rGST, rEIII and DENV on the induction of abnormal response of endothelial cells, including the induction of ROS (A, B), caspase-1 activity (C), IL-1β release (D), TNF-α release (E), soluble thrombomodulin release (F) at various time points were analyzed. * *P* < 0.05, ** *P* < 0.01, vs. respective rGST groups (n = 6; 3 experiments with 2 replicates).

**Fig. S2**


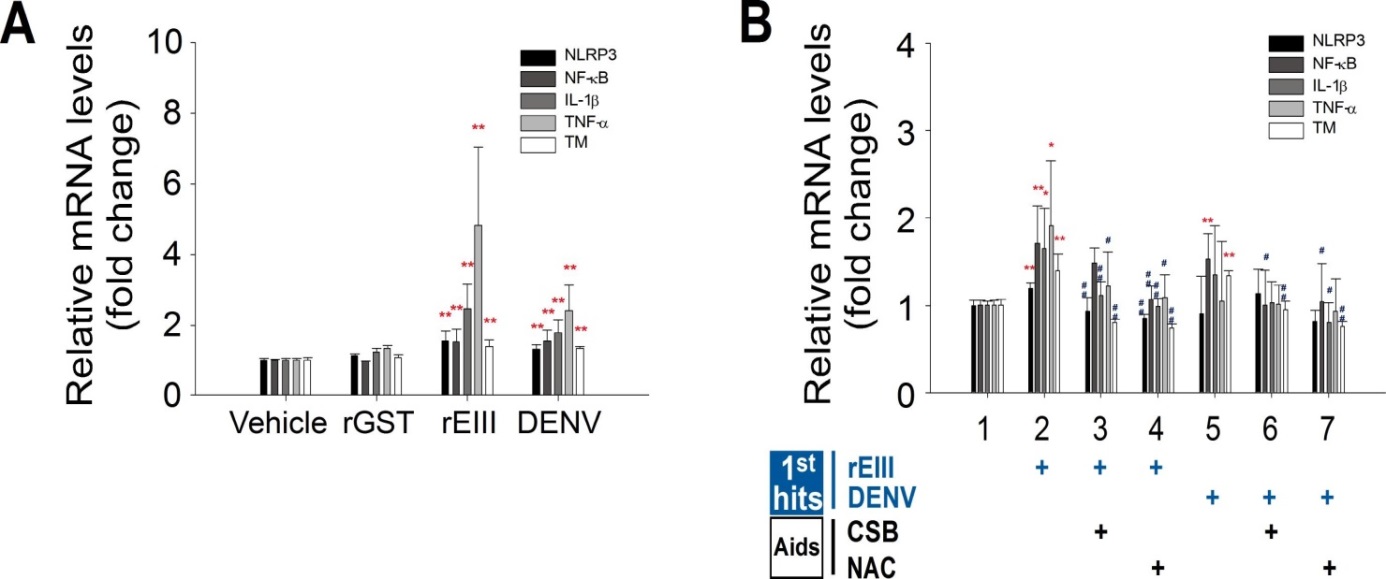


**Figure S2.** **Ameliorative effect of CSB and NAC treatments on rEIII-induced expressions of inflammation related genes in endothelial cells.** Quantitative real time polymerase chain reaction (qRT-PCR) analysis revealed that challenges of rEIII and DENV (DENV2 PL046) but not control protein rGST can elicit expressions of inflammation related genes, which include Nlrp3, NF-κb, IL-1β and TNF-α, in human HMEC-1 endothelial cells (A). Treatments of additional CSB (rEIII-binding GAG) and NAC (ROS scavenger), by contrast, markedly ameliorated proinflammatory changes that induced by rEIII and DENV in endothelial cells (B). * *P* < 0.05, ** *P* < 0.01, vs. respective vehicle groups; * *P* < 0.05, ** *P* < 0.01, vs. respective rEIII or DENV groups (n = 6; 3 experiments with 2 replicates).

**Fig. S3**


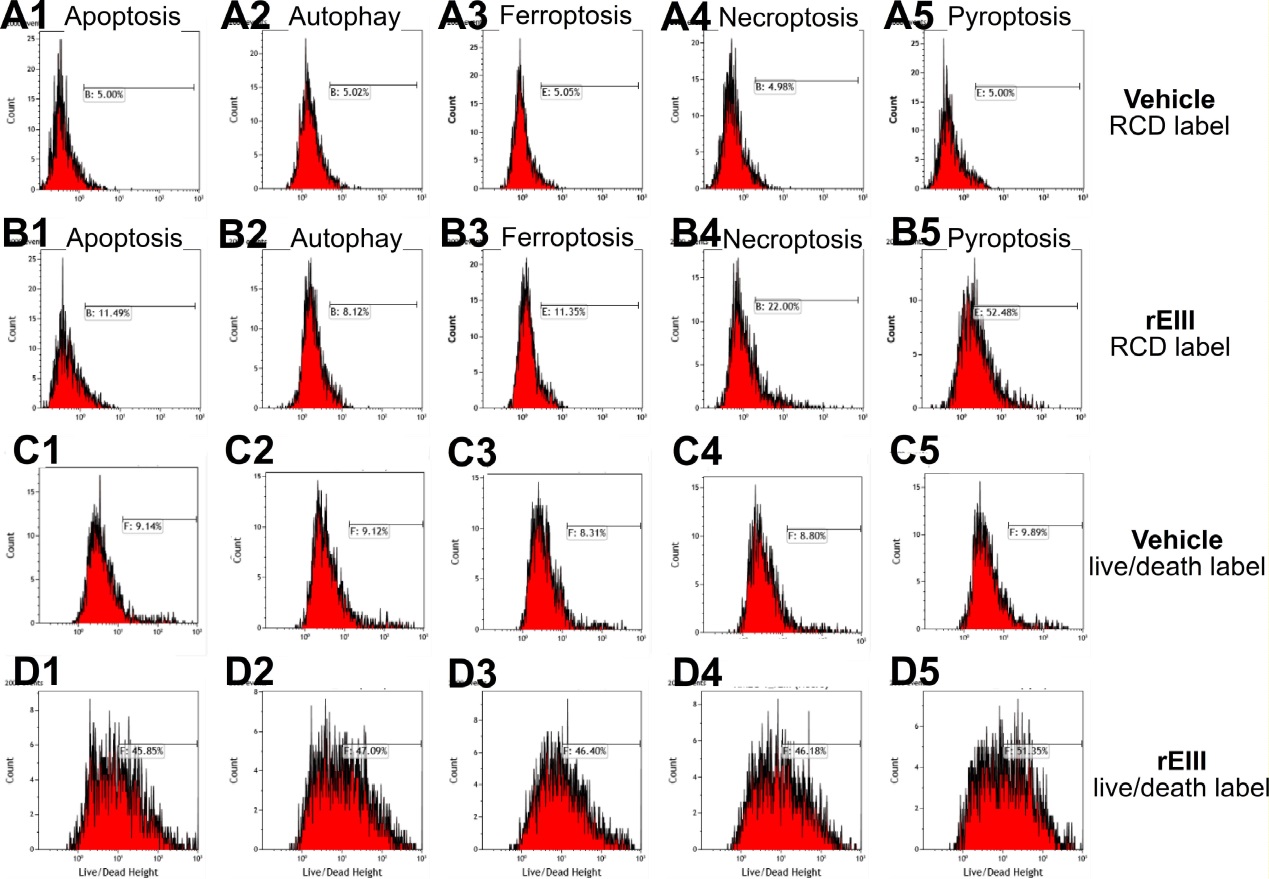


**Figure S3.** **Calculation of respective percentage of regulated cell death (RCD).** An example shows how respective RCD pathway percentages were determining by flow cytometry results. Because overlapping of detection wavelengths, it is not feasible to detect total 5 RCD pathways simultaneously in one cell-staining sample. Therefore, we performed double staining of respective RCD plus cell live and death statue staining (an internal control). For example, after endothelial cell samples were treated with vehicle (A, C) or rEIII (B, D), these endothelial cells were subjected to respective RCD (A, B) and cell-live/death status (C, D) staining. The respective increased cell death signal (e.g. apoptosis signal B1-A1; △apoptosis) was normalized (e.g. B1-A1/D1-C1; △ apoptosis/△total death cell) by increased death-cell population (e.g. D1-C1; △total death cell) of respective RCD staining. The sum of B1-A1/D1-C1, B2-A2/D2-C2, B3-A3/D3-C3, B4-A4/D4-C4 and B5-A5/D5-C5 was considered as 100%. As a result, the pyroptosis % was calculated as [B5-A5/D5-C5] / [(B1-A1/D1-C1) + (B2-A2/D2-C2) + (B3-A3/D3-C3) + (B4-A4/D4-C4) + (B5-A5/D5-C5)] × 100%, and is approximately 60%. Results showed in figure 3B were obtained through aforementioned formula using averaged results from triplicated samples of each group.

**Fig. S4**


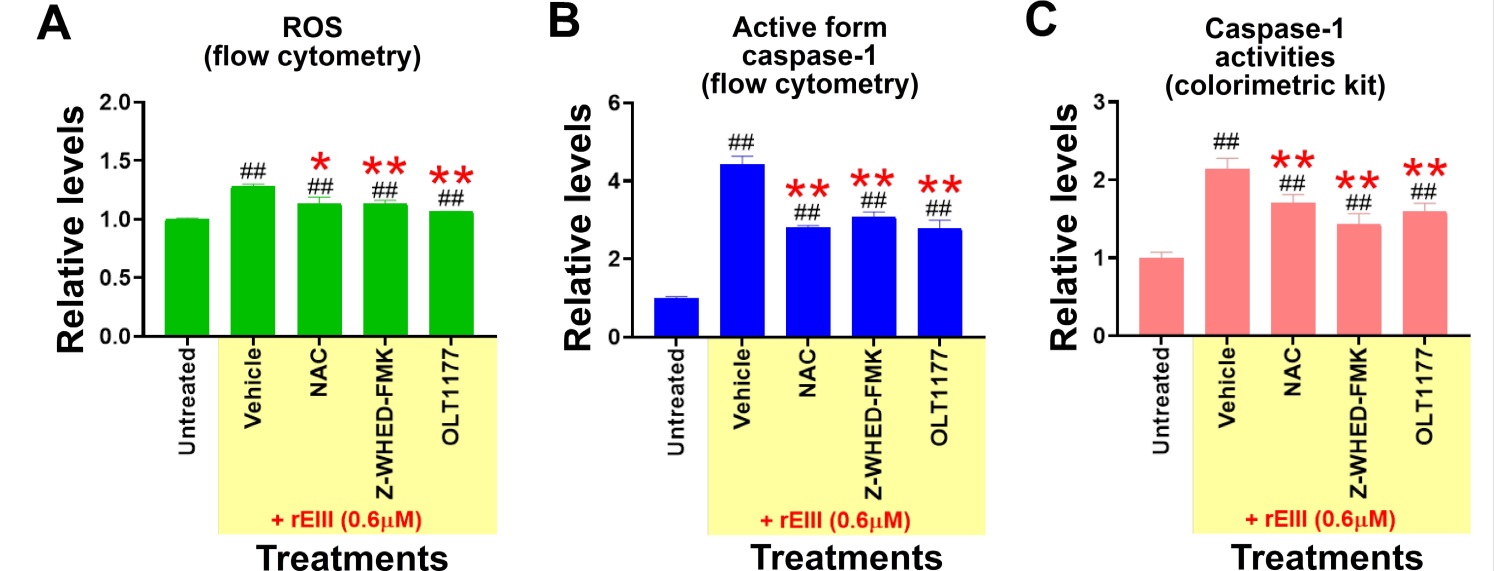


**Figure S4.** **Protection of endothelial cells from rEIII-induced pyroptosis under treatment with antioxidant NAC and Nlrp3 inflammasome inhibitors.** Treatment with antioxidant NAC (1 mM), Nlrp3 inhibitor OLT1177 (10 μM) and caspase 1 inhibitor Z-WHED-FMK (10 μM) rescued rEIII-induced endothelial cell ROS production (A), elevation of active form caspase-1 (B)(flow cytometry), and caspase-1 activity (C)(colorimetric kit). n = 6, ## *P* < 0.01, vs. respective untreated groups; **P* < 0.05, ***P* < 0.01, vs. respective vehicle groups.

**Fig. S5**


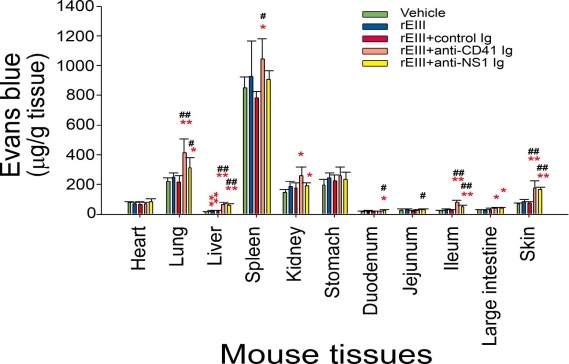


**Figure S5.** **Evans blue analysis in the two-hit mouse model.** Tissue retaining Evans blue levels were analyzed in the heart, lung, liver, spleen, kidney, stomach, duodenum, jejunum, ileum, large intestine and skin of mice after vehicle, rEIII, rEIII + control Ig, rEIII + anti-CD41 Ig and rEIII + anti-NS1 Ig challenges. * *P* < 0.05, ** *P* < 0.01, vs. respective vehicle groups, # *P* < 0.05, ## *P* < 0.01, vs. respective rEIII + control Ig groups (n = 6; 3 experiments with 2 mice per group).

**Fig. S6**


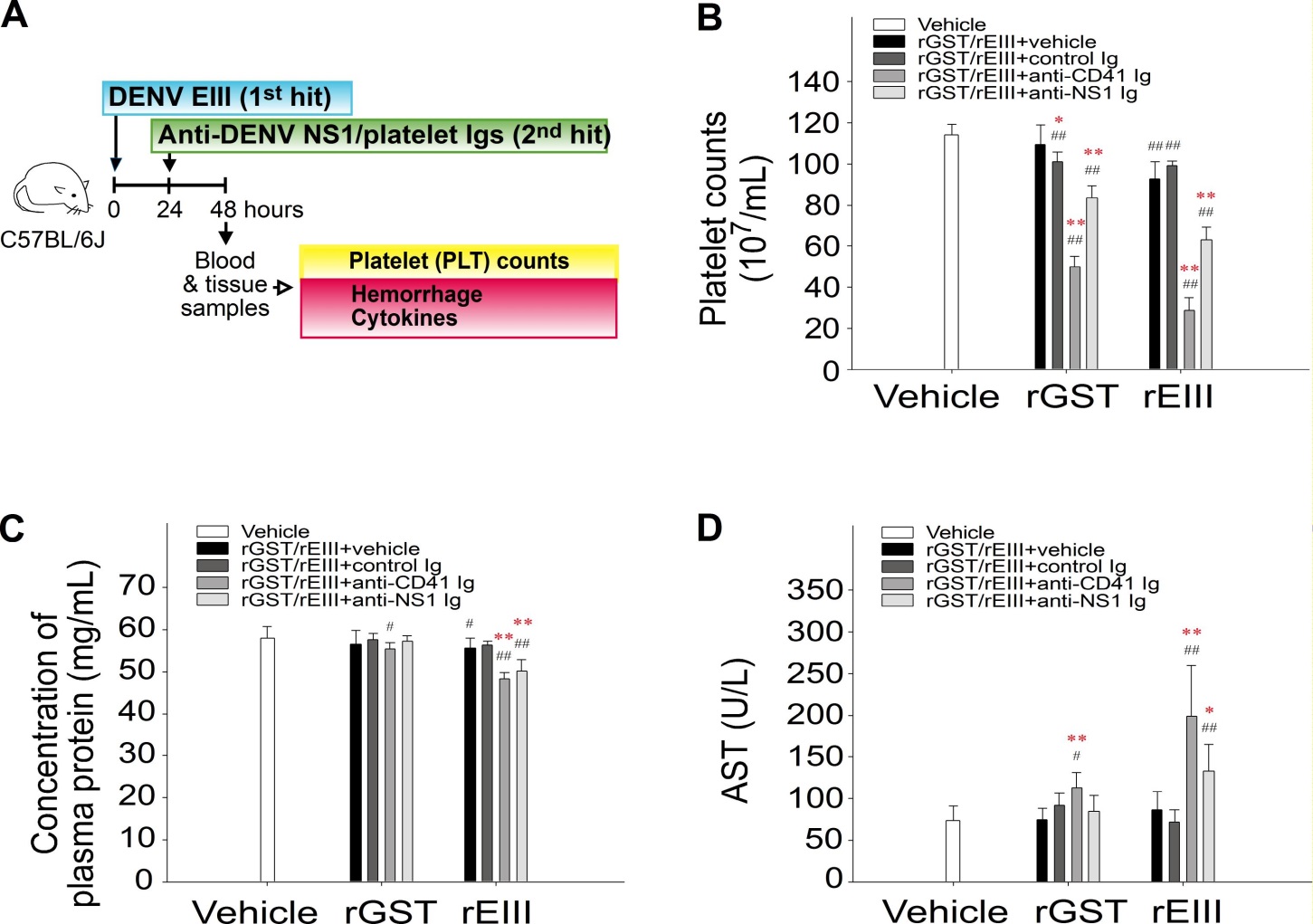


**Figure S6.** **Characterization of DHF-related manifestations in rEIII plus autoantibody two-hit mouse model.** The experimental outline was illustrated (A). Mice were injected with (rEIII) or without (rGST) rEIII (2 mg/kg; first hit) at time point 0 h and then were challenged by various antibody treatments (second hit; control Ig, anti-CD41 Ig, anti-NS1 Ig) 24 h later (A). Reduced levels of platelet counts (B) and plasma protein concentration (C), and the elevated aspartate transaminase (AST) levels (D) were measured at time point 48 h post first hit treatments. Results showed as mean +/- SD. (B-D) Red asterisks * *P* < 0.05, ***P* < 0.01 indicate significant exacerbation vs. respective rGST + control Ig (Ctrl Ig) or rEIII + Ctrl Ig groups; ^#^*P* < 0.05, ^##^*P* < 0.01 indicate significant exacerbation vs. vehicle groups. (B-D) n = 6 (three independent experiments with two replicates). The mouse drawing used in this figure was originally published in the Blood journal: Huang, H. S., Sun, D. S., Lien, T. S. and Chang, H. H. Dendritic cells modulate platelet activity in IVIg-mediated amelioration of ITP in mice. Blood, 2010; 116: 5002–5009. © the American Society of Hematology.

**Fig. S7**


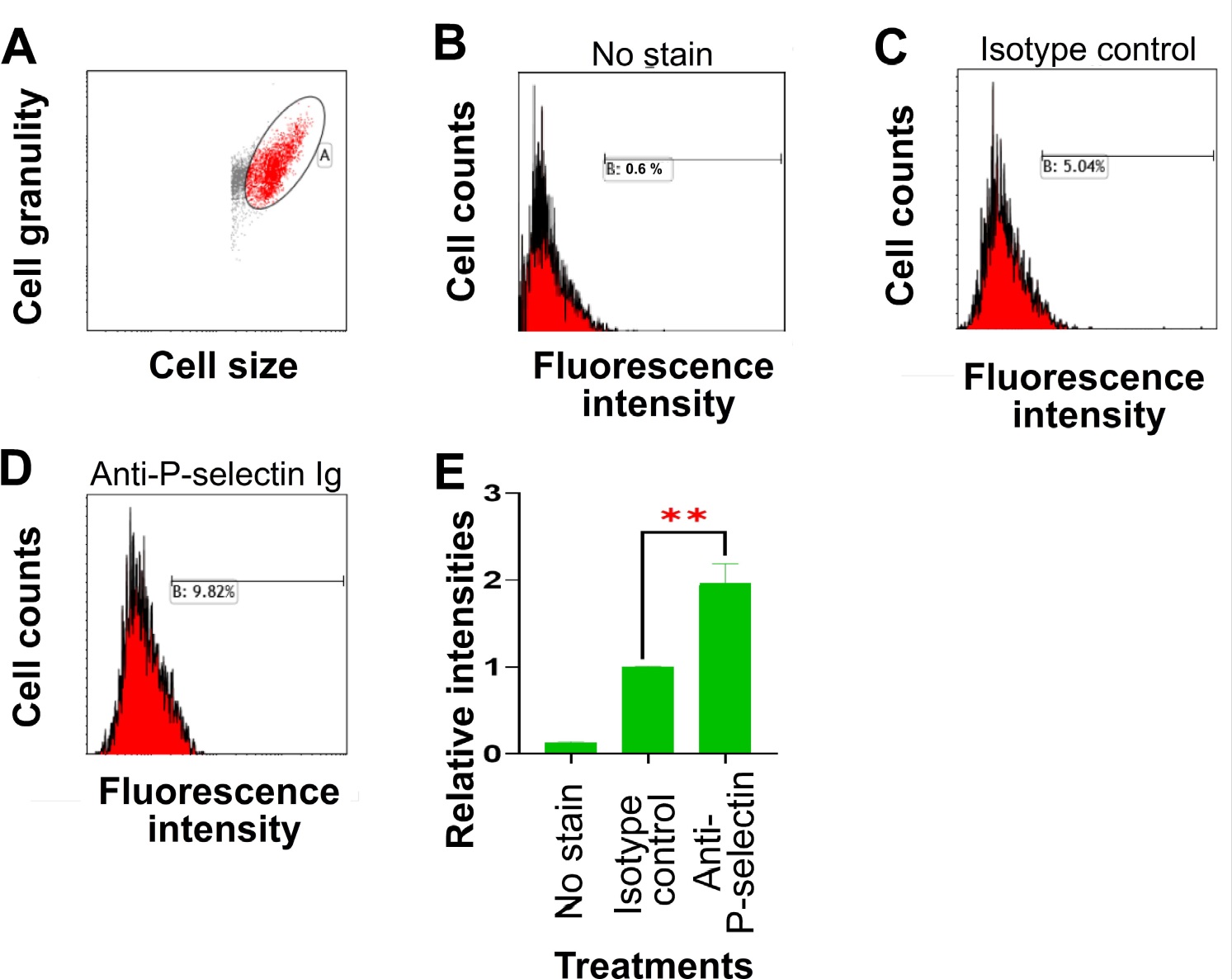


**Figure S7.** **Detection of surface P-selectin expression of HMEC-1 cells.** The flow cytometry gating (A-D; A, cell size/granularity; B, no stain; C, isotype control Ig; D, anti-P-selectin Ig), and relative fluorescence intensities of HMEC-1 cell surface P-selectin staining (E), in which the isotype control groups were normalized to 1-fold. n = 3, ** *P* < 0.01, vs. isotype control groups.

**Fig. S8**


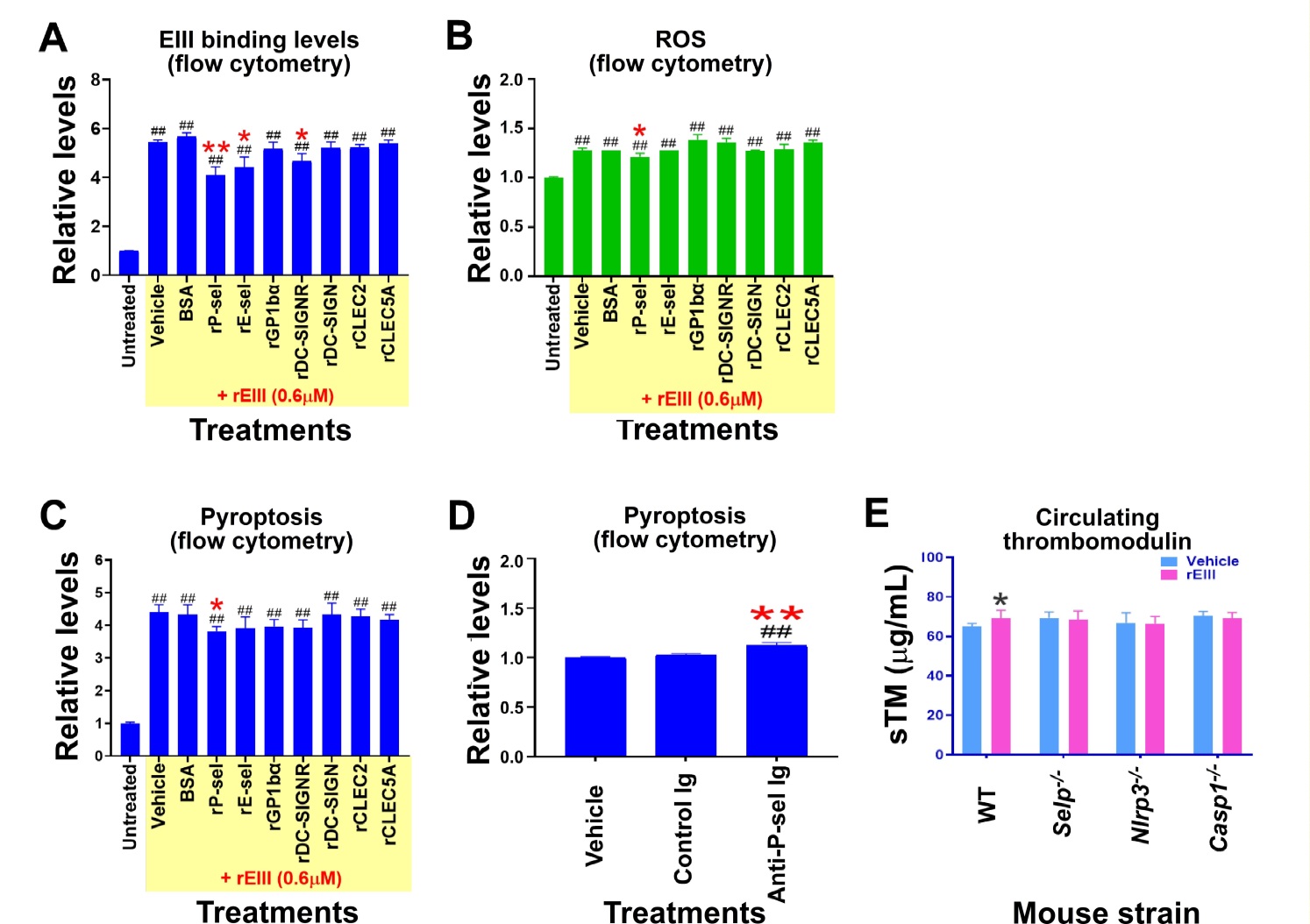


**Figure S8.** **P-selectin is a potential endothelial cell surface target of EIII.** Flow cytometry-based competition assay was performed. The EIII-HMEC-1 cell binding (A), EIII-induced HMEC-1 ROS production (B) and EIII-induced HMEC-1 active-form caspase-1^+^ cell population % (C, pyroptosis levels, Caspase-1 Assay, ImmunoChemistry Technologies), were competed by various soluble-form cellular receptors, including recombinant P-selectin (rP-sel), E-selectin (rE-sel), GP1bα, DC-SIGNR, DC-SIGN, CLEC2, CLEC5A, and negative controls vehicle and BSA (all proteins, 50 μg/mL). Pyroptosis levels of vehicle-, control Ig (50 μg/mL)-, and anti-P-selectin Ig (50 μg/mL)-treated HMEC-1 cells were also analyzed (D). Intravenous injections of rEIII (2 mg/kg) on the induction of mouse circulating soluble thrombomodulin (sTM) levels (24 h after EIII injections; thrombomodulin ELISA kit, Abcam) are indicated (E). ## P < 0.01, significant increase vs. untreated groups; * *P* < 0.05, ** *P* < 0.01, significant decrease vs. vehicle control groups (A-C); ** *P* < 0.01, vs. vehicle control groups, ## *P* < 0.01, vs. control Ig groups (D); * *P* < 0.05, vs. respective vehicle control groups (E).

**Fig. S9**


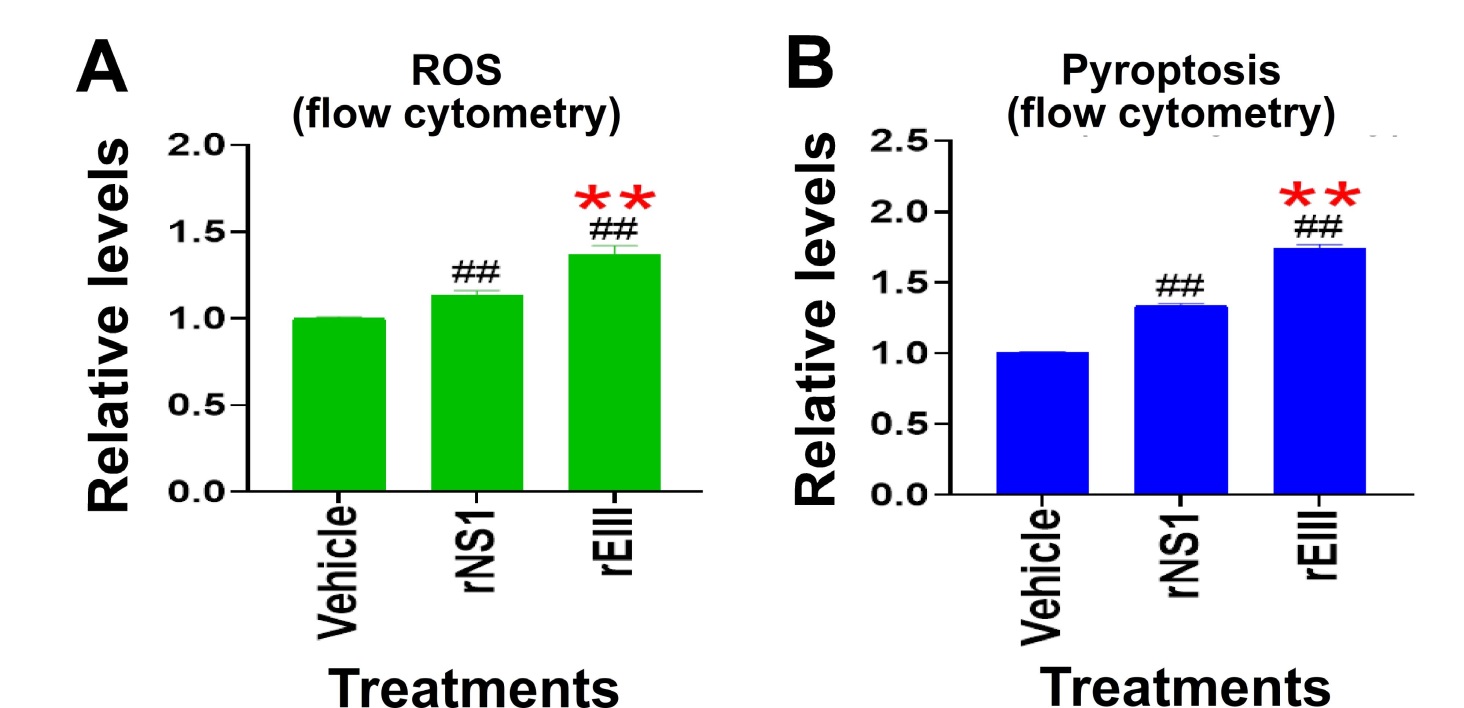


**Supplementary Figure 9. Treatments of DENV rNS1 and rEIII increase HMEC-1 endothelial cell ROS and pyroptosis levels.** Flow cytometry analysis on the percentage of DENV rNS1 and rEIII-induced ROS (A) and pyroptosis (B) in total population. Dosage used, rNS1, 0.6 μM, rEIII, 0.6 μM, both are adjusted approximately reached to the clinically detected levels. n = 6. ## *P* < 0.01, vs. respective vehicle groups; ** *P* < 0.01, vs. respective rNS1 groups.

**Supplemental Table**

**Table S1**. Primers used in qRT-PCR analyses.

| **Genes** | **Primers** | **Fragment size (bp)** |
| --- | --- | --- |
| **Nlrp3** | 5’- GCCTGTTCTCATGGATTGGT-3’  5’- CCGAATGTTACAGCCAGGAT-3’ | 177 |
| **NF-κB** | 5’- CTGGAAGCACGAATGACAGA-3’  5’- TGAGGTCCATCTCCTTGGTC-3’ | 172 |
| **IL-1β** | 5’- GCTGAGGAAGATGCTGGTTC-3’  5’- TCCATATCCTGTCCCTGGAG-3’ | 240 |
| **TNF-α** | 5’-TCCTTCAGACACCCTCAACC-3’  5’- AGGCCCCAGTTTGAATTCTT-3’ | 173 |
| **Thrombomodulin** | 5’-TACGGGAGACAACAACACCA-3’  5’- AAGTGGAACTCGCAGAGGAA-3’ | 180 |
| **β-actin** | 5’- ATCTGGCACCACACCTTCTA-3’  5’- GGGTGTTGAAGGTCTCAAAC-3’ | 136 |
